# Dr. T. F. Allen on Homœopathy

**Published:** 1890-09

**Authors:** E. W. Berridge

**Affiliations:** London, England


### DR. T. F. ALLEN ON HOMŒOPATHY.

E. W. BERRIDGE, M. D., LONDON, ENGLAND.

In the May number of THE HOMŒOPATHIC PHYSICIAN Dr. T. F. Allen publishes his protest against recent criticisms, insisting upon the accuracy of his definition of a homœopath, claiming that he has always practiced and taught "straight Homœopathy," and accusing some of those who profess to be

Hahnemannians of departure from the faith. Such a protest demands the most serious investigation.

(1.) Dr. T. F. Allen argues that a Christian is one who professes himself to be one, publicly unites with those who profess a similar faith, and does the best he can to live the life. And that, similarly, a homœopath is one who believes Homœopathy to be the best way of curing the sick, joins a homœopathic society, and does his best to practice Homœopathy. With this definition no one can find much fault, but the question is, *do they all strive to "live the life"?* If a professing Christian should get drunk, he undoubtedly commits an unchristian act; but no one, except a Pharisee, would deprive him of church membership on that account, always provided that he was truly penitent, and resolved never to fall from grace again.

So with Homœopathy. No man can do more than his best, *but does he always do his best?* Here are three imaginary cases of renal colic. Dr. A. treats one. He is a strict Hahnemannian, and possessed of the knowledge of how to elicit the patient's symptoms, and how to select the remedy from the materia medica. He prescribes the *simillimum*, and cures his patient homœopathically. He is a true homœopath, and a successful one.

Dr. B. is also a Hahnemannian, but of small experience. He honestly endeavors to select the *simillimum*, but fails. The patient is in acute suffering, and his knowledge of Homœopathy is exhausted. He knows that by an opiate or an anæsthetic he can give temporary relief, and, perhaps, concludes that whatever ill effects may arise they will be less than the ill effects of letting the patient continue to suffer. So he prescribes an allopathic or antipathic palliative. Is he a true homœopath? The answer to this question depends entirely on his future line of practice. If he admits that Homœopathy is a law of nature, and therefore infallible, though he himself is not infallible in applying it; if he subsequently studies his case again and again, and asks the help of those of longer and wider experience, that he may see where his error lay, and if he firmly resolves that he will endeavor to do better next time, then he is a true homœ-

opath, who has stumbled on the road, but recovered his footing. But if he declares, perhaps, from this one case, that there are cases where Homœopathy is of no avail, and where allopathy must be resorted to, then he is no longer a homœopath, but a traitor and a renegade.

Dr. C. also treats a case of renal colic, but, instead of endeavoring to select the *simillimum*, he at once gives *Morphia*. This man, I maintain, is not a homœopath; he is simply a base pretender. And no membership in a professed homœopathic society can make him a homœopath, any more than church membership can make a confirmed drunkard a Christian.

(2.) Dr. T. F. Allen says he finds "the most exclusive practitioners of our art reporting cures made with the highest potencies of a drug which has never been proved, whose indications are wholly clinical." This he calls "high potency allopathy." But as "allopathy" means the prescribing of drugs which produce symptoms different from those of the patient, how can he demonstrate that the cure is allopathic, if there is no proving of the remedy? And will he seriously declare that cures by high potencies can ever be allopathic? It is easily tested. Let him persuade some allopathic friend to give for twelve months the same medicines which he would usually prescribe, but in a high potency; we shall soon see the result. Again, is not Dr. T. F. Allen rather "previous" in accusing any of us of prescribing unproved medicines? The *Encyclopœdia* does not contain everything, as I myself, as well as others, happen to have fragmentary provings of medicines which are as yet known to the profession generally by their clinical effects only. All these, I trust, will be published in due time. But, after all, is the prescribing upon clinical symptoms really the unpardonable sin? If so, then Hahnemann himself is lost beyond redemption. If Dr. T. F. Allen will examine any of Bœnninghausen's *Repertories* he will find countless symptoms, not to be found as yet in our materia medica. It is surely not unreasonable to conclude that a large proportion of these are clinical. And yet Hahnemann said he preferred Bœnninghausen's *Repertories* to all others.

(3.) Dr. T. F. Allen declares that "it is absolutely true that every physician in large practice is obliged to use other than homœopathic methods in the treatment of the sick." I think some of the veterans of the I. H. A. will have a word to say on this subject. In the meantime I can reply for two Hahnemannians. It is nearly thirty years ago that I became acquainted with my *true* friend and preceptor, the late Dr. David Wilson, and from the day of our first acquaintance to the day of his death we maintained a firm and unbroken friendship. Our belief in Hahnemann was the same, as was also our method of selecting the remedy by means of the *Materia Medica* and *Repertory*, and he used to tell me that he looked upon me as the one who would take his place after he had left this world. I, therefore, can speak with authority on his mode of practice, and I assert that he used frequently to declare *that he had never once found Hahnemann's teaching to be wrong, and hence never had occasion to diverge from it.* For my own part, I declare the same thing. On another point also I must dispute Dr. T. F. Allen's assertion. He says that these departures from Homœopathy are necessary, "not for their cure, but sometimes for their palliation when they cannot be cured," and that "this practice will continue until our *Materia Medica* is so complete that every patient will be cured." Dr. T. F. Allen's argument is that in incurable cases Homœopathy is insufficient. On the contrary, I have found by experience that Homœopathy relieves these cases, and promotes *euthanasia*, far better than allopathy. I have had opportunities of comparing the two methods, and I can, therefore, speak with confidence. And, further, even were our *Materia Medica* complete, there would still be some cases which would prove fatal through failure of the vital powers in old age, or deeply-rooted disease of important organs.

(4.) Dr. T. F. Allen declares that "for twenty-five years and more I have faithfully and conscientiously practiced and taught straight Homœopathy." I am pleased to hear it, and only regret that if such is the case he has not given the support of his name to the I. H. A. But alas! In THE HOMŒOPATHIC PHYSICIAN, vol. VII, p. 398, I read a paper from him containing the following words: "He, who, in these days will not wash out with distilled water and one-five thousandth of a grain of Corrosive Mercury a fresh case of gonorrhœa, and cure his erring brother in twenty-four to forty-eight hours, must give up the treatment of such diseases." If this can be done, physicians ought to be able to make a fortune out of the magical treatment of this disease, and the fact that they do not is a strong proof that the charm will not work. For my own part I never give injections for gonorrhœa, having seen too much harm arise from them, and I never treat them otherwise than homœopathically, and I have always been successful. But, apart from the question of the results of the two methods of treatment, another question arises, which I hope Dr. T. F. Allen will answer. *What has this routine treatment of fresh gonorrhœa to do with "straight Homœopathy."*

